# A new mouse model for a high-risk AMD variant, *ARMS2 ^A69S^*

**DOI:** 10.64898/2026.10.02.756275

**Published:** 2026-10-16

**Authors:** Navdeep Gogna, Gayle B. Collin, Lillian F. Hyde, Lisa Stone, Ravi Pandey, Victoria E. Kennedy, Mark P. Krebs, Juergen K. Naggert, Patsy M. Nishina

## Abstract

Age-related macular degeneration (AMD) is the leading cause of irreversible central vision loss in older adults. The highest genetic risk for AMD maps to Chromosome 10q26. However, due to strong linkage disequilibrium between two genes, *ARMS2* and *HTRA1* located in this region, it has been difficult to determine whether one or both genes contribute to the AMD-disease risk. Functional studies of *ARMS2* have been hampered due to the lack of animal models, as orthologs are only found in the higher primates and the gene is absent in rodents. To model *ARMS2* biology *in vivo* , we used CRISPR-Cas9 to insert the complete human *ARMS2* gene carrying the A69S risk variant (rs10490924), into a region of homology between humans and mice, on mouse Chromosome 7, 3.5 kb upstream of *Htra1* , preserving its native promoter, spacing, and orientation. We also generated a mouse model bearing its wild-type counterpart *ARMS2* ^*A69*^ ( *ARMS2* ^*WT*^ ). RNA analysis revealed tissue- and cell-type-specific patterns of *ARMS2* expression with enrichment in neural tissues, testis, and eye, and within the eye, in retinal horizontal cells and the retinal pigment epithelium (RPE). *ARMS2* expression was consistent with human expression data and unaltered by the A69S mutation. By 12 months of age, *ARMS2* ^*A69S*^ mice developed AMD-relevant phenotypes including increased fundus spots, A69S-specific thinning of the outer nuclear layer, and reduced scotopic ERG amplitudes. Transcriptomic profiling of postnatal day 14 retina and RPE-enriched fractions revealed reduced mitochondrial oxidative-phosphorylation genes, corroborated by reduced retinal mtDNA copy number and lower maximal respiration, alongside preliminary results that suggest sphingolipid remodeling in the RPE. This humanized model links the *ARMS2* A69S variant to mitochondrial dysfunction and provides an *in vivo* platform for defining AMD mechanisms and testing therapies.

## Full Text Availability

The license terms selected by the author(s) for this preprint version do not permit archiving in PMC. The full text is available from the preprint server.

